# High-pressure phase transition in 3-D printed nanolamellar high-entropy alloy by imaging and simulation insights

**DOI:** 10.1038/s41598-024-67422-x

**Published:** 2024-07-16

**Authors:** Andrew D. Pope, Wen Chen, Hangman Chen, Penghui Cao, Armenuhi Yeghishyan, Maksym Zhukovskyi, Khachatur Manukyan, Yogesh K. Vohra

**Affiliations:** 1 Department of Physics, University of Alabama at Birmingham, Birmingham, AL 35294 USA; 2 Department of Mechanical and Industrial Engineering, University of Massachusetts Amherst, Amherst, MA 01003 USA; 3 Mechanical and Aerospace Engineering, University of California, Irvine, CA 92697 USA; 4 Nuclear Science Laboratory, Department of Physics and Astronom, University of Notre Dame, Notre Dame, IN 46556 USA; 5 Notre Dame Integrated Imaging Facility, University of Notre Dame, Notre Dame, IN 46556 USA

**Keywords:** Engineering, Materials science, Nanoscience and technology, Physics

## Abstract

We report on the high-resolution imaging and molecular dynamics simulations of a 3D-printed eutectic high-entropy alloy (EHEA) Ni_40_Co_20_Fe_10_Cr_10_Al_18_W_2_ consisting of nanolamellar BCC and FCC phases. The direct lattice imaging of 3D-printed samples shows the Kurdjumov–Sachs (K–S) orientation relation {111} FCC parallel to {110} BCC planes in the dual-phase lamellae. Unlike traditional iron and steels, this alloy shows an irreversible BCC-to-FCC phase transformation under high pressures. The nanolamellar morphology is maintained after pressure cycling to 30 GPa, and nano-diffraction studies show both layers to be in the FCC phase. The chemical compositions of the dual-phase lamellae after pressure recovery remain unchanged, suggesting a diffusion-less BCC–FCC transformation in this EHEA. The lattice imaging of the pressure-recovered sample does not show any specific orientation relation between the two resulting FCC phases, indicating that many grain orientations are produced during the BCC–FCC phase transformation. Molecular dynamics simulations on phase transformation in a nanolamellar BCC/FCC in K–S orientation show that phase transformation from BCC to FCC is completed under high pressures, and the FCC phase is retained on decompression aided by the stable interfaces. Our work elucidates the irreversible phase transformation under static compression, providing an understanding of the orientation relationships in 3-D printed EHEA under high pressures.

## Introduction

Phase transitions in crystalline solids are critical phenomena across various materials, including metals^1,2^, alloys^3–5^, semiconductors^6,7^, and ceramics^8–10^. These transitions significantly influence the mechanical, optical, electrical, and thermal properties of these materials^5,11–14^. In metals and alloys, the transition between body-centered cubic (BCC) and face-centered cubic (FCC) crystal structures and its reverse process stand out for their significance^15,16^. The reversible FCC–BCC phase transitions are utilized to create diverse material classes, such as steels^4^, catalysts^12,17^, and high enthalpy alloys^18,19^, for varied applications.

The BCC to FCC transition has been extensively investigated in the Pd–Cu model alloys, yet the atomistic mechanism of this transition remains not well understood^17,20–22^. Molecular dynamics (MD) simulation indicated that the FCC phase should nucleate at an interface involving a slip of edge dislocation of 1/6a_BCC_[01¯1] on (011) planes, followed by growth through the slip of newly formed screw dislocation planes and adjustment to FCC lattice by volume expansion^23^. These simulations also revealed that a minimal energy difference between interface and surface energies drives the nucleation of the FCC phase, thus enabling such transition at the BCC–FCC interface with the Nishiyama–Wassermann (N–W) orientation^23^. In contrast, elevated interface energy is a barrier to the phase transition at interfaces exhibiting the Kurdjumov–Sachs (K–S) relationship^23^.

The BCC to FCC transition also occurs during the casting of compositionally complex alloys, such as CoCrFeNiMn and AlCoCrFeNi^24–26^. A classic dual-phase microstructure emerging from such transformation at slow cooling rates is the Widmanstätten structure, where FCC colonies grow from grain boundaries into the parent BCC phase^27^. High cooling rates during casting can entirely suppress the growth of the FCC phase^27^. The annealing of as-cast BCC alloys at ~ 1000 K triggers a BCC to FCC transition^25^. The KS orientation relationship is observed between the BCC and the newly formed FCC phase, with FCC grains forming near dendritic boundaries. This suggests that dislocations at grain boundaries act as nucleation sites for the FCC phase^25^.

Additive manufacturing techniques, such as laser-powder bed fusion (3D-printing), produce far-from-equilibrium micro/nanostructures in compositionally complex alloys, yielding mechanical properties superior to conventional as-cast alloys^18,28^. For example, the eutectic high-entropy alloy (EHEA) Ni_40_Co_20_Fe_10_Cr_10_Al_18_W_2_ exhibits nanolamellar BCC and FCC phases under ambient conditions, with a yield strength as high as 1.49 GPa and tensile strain exceeding 14%^29^. Early high-pressure studies on nanolamellar structures in EHEA have documented a BCC to FCC phase transition under high pressures^30^. However, the detailed mechanism of this phase transition remains elusive due to the absence of high-resolution imaging of pressure-recovered samples. Questions such as whether the nanolamellar structure is preserved during transformation at high pressures or if there is any change in the chemical composition of the nanolamellar phases due to the phase change from BCC to FCC remain unanswered. Additionally, establishing the orientation relationship between the pressure-induced FCC phase and the as-printed FCC phase is crucial. To bridge these knowledge gaps, we employed high-resolution electron microscopy to image the pressure-induced BCC to FCC phase transition in the additively manufactured EHEA Ni_40_Co_20_Fe_10_Cr_10_Al_18_W_2_. We compressed the sample beyond the critical phase transition pressure of 9 GPa and then recovered it for detailed imaging analysis. Alongside, we conducted nonequilibrium MD simulations to explore the underlying mechanism of the BCC to FCC phase transition.

## Results

### High-resolution studies of 3-D printed EHEA

**Figure 1:**
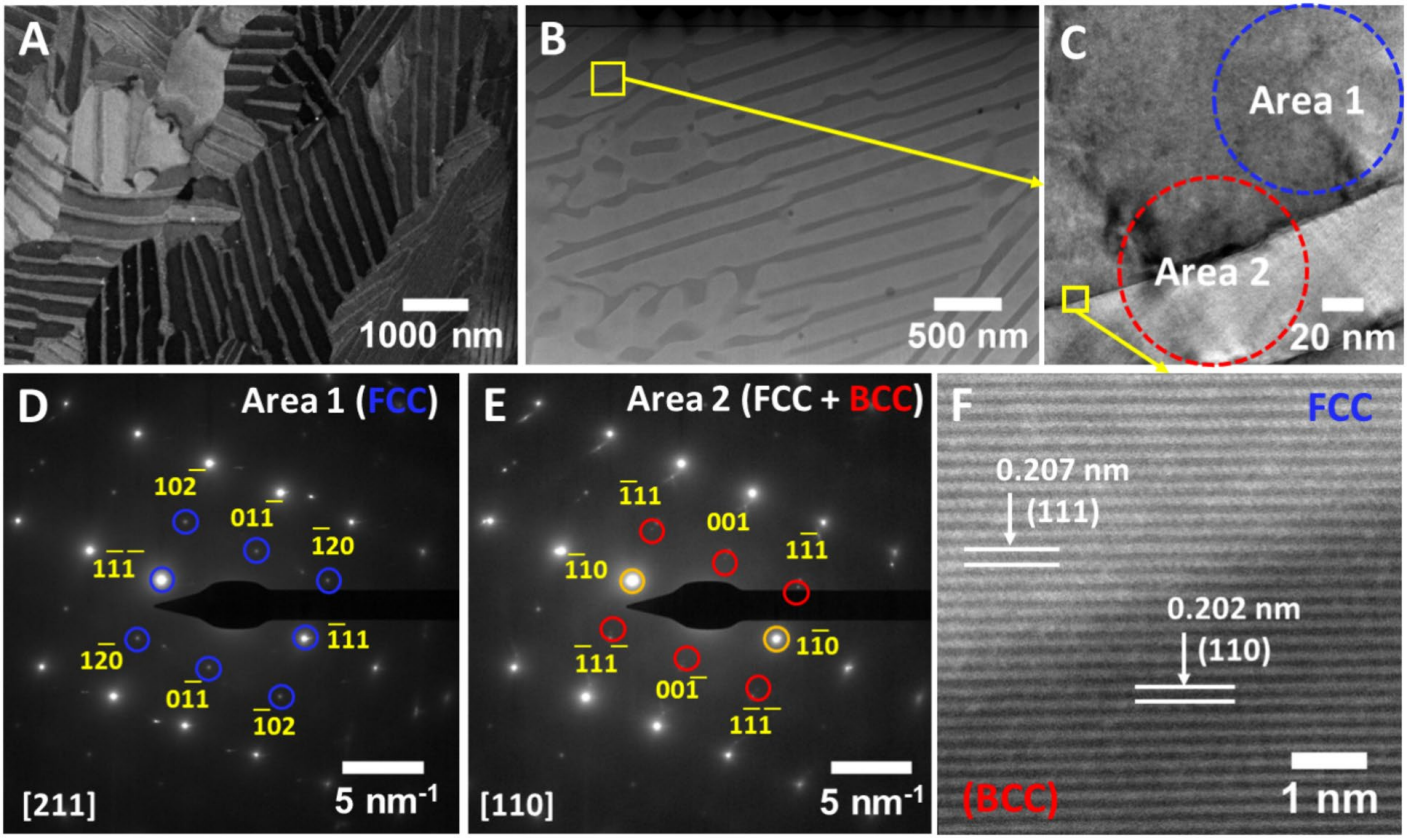
Structure and morphology of as-printed Ni_40_Co_20_Fe_10_Cr_10_Al_18_W_2_ EHEA: A backscatter SEM image of the polished and gallium ion-etched sample surface (**A**), a HAADF STEM image of the specimen showing two phases with distinctive contrasts (**B**), a magnified bright-field TEM image of a region with two phases (**C**), SAED patterns taken from Area 1 (**D**) and Area 2 (**E**) highlighted on the panel C showing the presence of FCC and FCC + BCC phases, respectively, as well as a high-resolution HAADF STEM image of the FCC/BCC phase interface with (111) and (110) orientations, respectively (**F**).

Figure 1 summarizes the structure and morphology of as-printed EHEA Ni_40_Co_20_Fe_10_Cr_10_Al_18_W_2_. The eutectic colonies showing alternate BCC and FCC nanolamellae are clearly visible in Fig. 1A. The high-angle annular dark-field scanning transmission electron microscopy (HAADF-STEM) image of nanolamellae shows dark (BCC phase) and light (FCC phase) regions. Analysis of multiple backscatter scanning electron microscopy (SEM) images shows the average thickness of BCC nanolamellae is 80 nm while the average thickness of FCC nanolamellae is 200 nm (Supplementary Information, Figure S1). Such measurements also show that BCC grains have a wider thickness distribution.

A magnified bright-field TEM image shows a well-defined boundary between two phases (Fig. 1C). Two areas for electron diffraction are also marked in Fig. 1C. The selected area electron diffraction (SAED) pattern taken from “Area 1” (Fig. 1D) shows characteristic reflections for an FCC grain. In contrast, the SAED pattern from the interface between “Area 1” and “Area 2” (Fig. 1E) shows a complex diffraction pattern exhibiting diffraction spots for both FCC and BCC phases. The high-resolution lattice imaging in Fig. 1F reveals the orientation relationship between the dual-phase nanolamellae, with {111} FCC planes parallel to {110} BCC planes, corresponding to the K–S orientation relation.

### High-pressure diamond anvil cell studies and sample recovery

**Figure 2:**
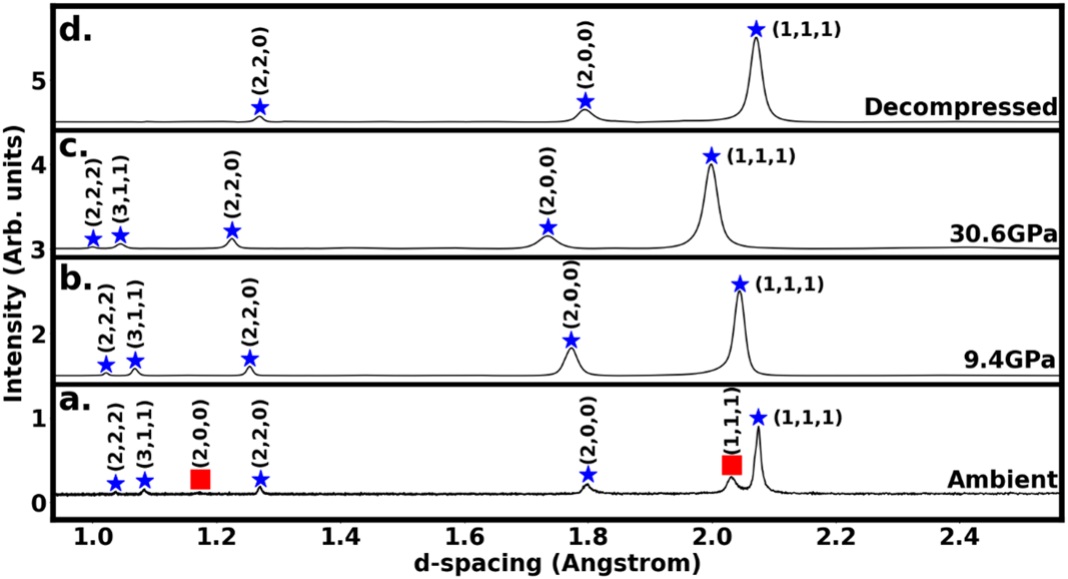
X-ray diffraction patterns of Ni_40_Co_20_Fe_10_Cr_10_Al_18_W_2_ EHEA in a diamond anvil cell (**a**) at ambient condition showing both BCC and FCC phases, (**b**) showing complete transformation to FCC phase, (**c**) at 30.6 GPa showing stability of the FCC phase to the highest pressure, and (**d**) after decompression to ambient pressure showing retention of the FCC phase. Red symbols mark the BCC peaks, while the FCC are marked by blue symbols. The pressures were measured by the ruby fluorescence technique.

Figure 2 shows the angle dispersive x-ray diffraction (XRD) patterns from EHEA Ni_40_Co_20_Fe_10_Cr_10_Al_18_W_2_ at various pressures in a diamond anvil cell. The ambient pressure XRD in Fig. 2a is indexed to a phase mixture consisting of a BCC phase with lattice parameter *a* = 2.871 Å and an FCC phase with a lattice parameter *a* = 3.591 Å. The sample is a single-phase FCC at pressures above 9 GPa as shown in Fig. 2b and c shows FCC XRD pattern at 30.6 GPa with a measured lattice parameter *a* = 3.456 Å (volume compression of 11%). On the release of pressure, the single-phase FCC is retained on decompression to ambient pressure, as shown in Fig. 2d, and the measured lattice parameter for the decompressed FCC spectrum is *a* = 3.588 Å. The slight difference in the ambient pressure and decompression FCC lattice parameters corresponds to a residual compressive strain of 0.3% and is within the experimental error of our measurements.

### High-resolution imaging of pressure recovered sample

The metallic gasket holding the EHEA Ni_40_Co_20_Fe_10_Cr_10_Al_18_W_2_ sample was carefully removed from the diamond anvil cell after the high-pressure excursion to 30.6 GPa. The sample is mounted in epoxy resin and carefully polished (Figure S2). Focus-ion beam milling is used to lift out multiple 50 nm thickness and several microns in length lamellas for high-resolution TEM imaging (Figure S2).

**Figure 3:**
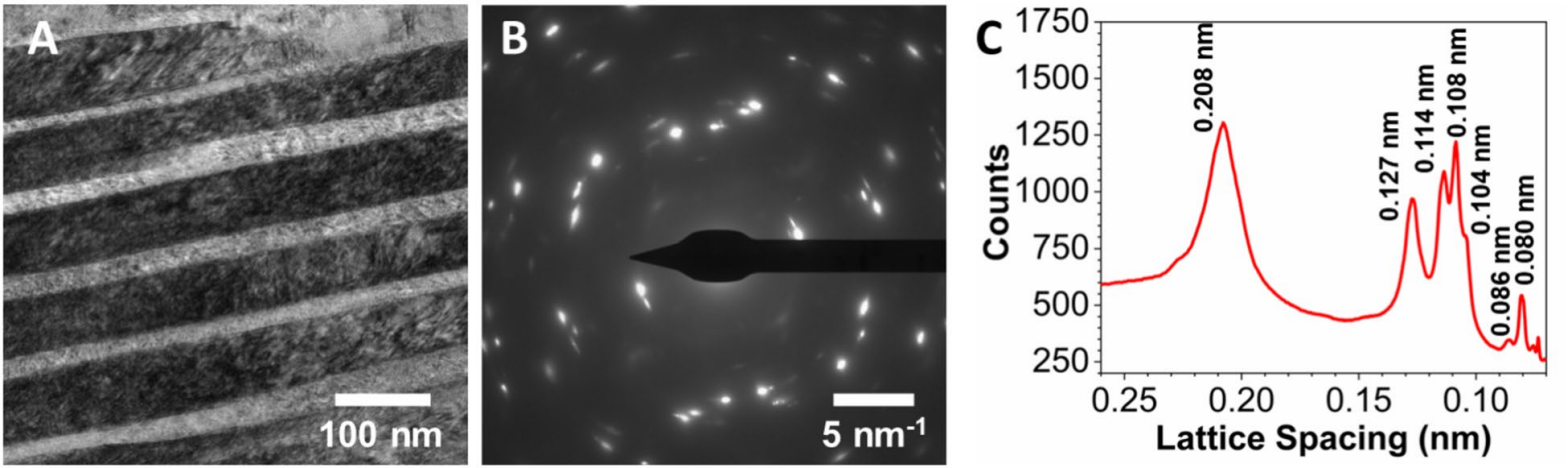
Structure and morphology of compressed Ni_40_Co_20_Fe_10_Cr_10_Al_18_W_2_ EHEA sample: BF TEM image of a laminated area of the specimens (**A**), a SADP taken from this area (**B**), and a profile obtained from SADP (**C**).

It is remarkable to note that the nanolamellar morphology of the EHEA Ni_40_Co_20_Fe_10_Cr_10_Al_18_W_2_ sample is retained after compression to pressure as high as 30.6 GPa (Fig. 3A). A representative SAED pattern from this region is shown in Fig. 3B, and the corresponding diffraction profile is shown in Fig. 3C. We should note that SAED patterns show bright spots and ring-shaped features, indicating the presence of both textured “coarse-grain” and fine polycrystalline structures. However, comparing the measured lattice spacing (Table S1) on the pressure recovered sample with our ambient pressure values based on FCC lattice parameter *a* = 3.591 Å shows an excellent agreement and provides conclusive evidence that the sample is a single-phase FCC material.

**Figure 4:**
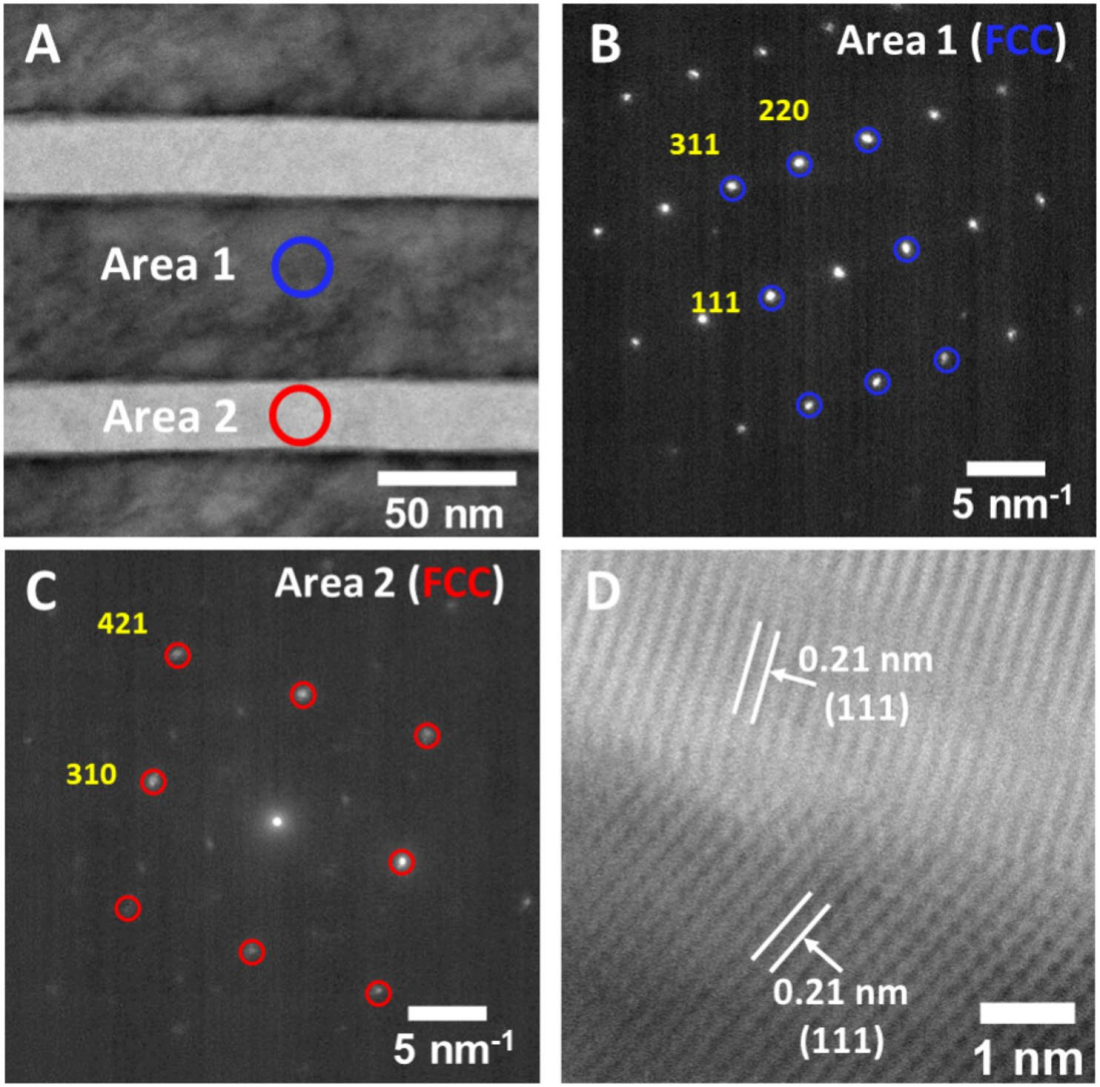
Structure of compressed Ni_40_Co_20_Fe_10_Cr_10_Al_18_W_2_ EHEA: HAADF STEM image of the specimen showing two phases (**A**), nano-diffraction patterns taken from Area 1 (**B**) and Area 2 (**C**) highlighted on panel A showing the presence of FCC for both phases and a high-resolution BF STEM image taken at the interface of two FCC phases (**D**).

Furthermore, nano-diffraction measurements conducted on individual nanolamellae conclusively prove that both layers are in the FCC phase, as indicated in Fig. 4. Area 1, measured on a darker layer imaged in STEM mode (Fig. 4A), clearly shows bright diffraction spots indexed to the FCC phase (Fig. 4B). The nano-diffraction pattern measured from Area 2 also shows bright spots for the FCC phase. However, such patterns also contain many spots with much less intensity, confirming the ultra-file polycrystalline nature of the pressure-induced FCC phase.

We further employed high-resolution STEM imaging to investigate the orientation relationship between the two FCC phases within the recovered sample. Examination of various regions along the nanolamellae boundary revealed the absence of a specific orientation relationship between the two FCC lattices, as multiple variants of FCC structures emerge from the parent BCC lattice (Fig. 4D). Imaging individual grains within the newly formed FCC nanolamellae presents a significant challenge due to their minuscule dimensions and indistinct grain boundaries (Figure S3). Nevertheless, a representative Fast Fourier Transform (FFT) image of a newly formed FCC nanolamellae displays multiple overlapping spots from the lattice planes of different FCC crystallites, indicating grains are randomly oriented to each other. In contrast, an FFT image of an untransformed FCC grain highlights the single-crystalline nature of this phase (Figure S3). Our observations indicate that the pressure induced nucleation of FCC phase starts at multiple locations within the BCC nanolamellae and is not confined to the interface region between BCC and FCC where orientation relationship exists. We suggest a case of homogenous nucleation of FCC within a BCC nanolamellae under high pressures.

### MD simulations

**Figure 5:**
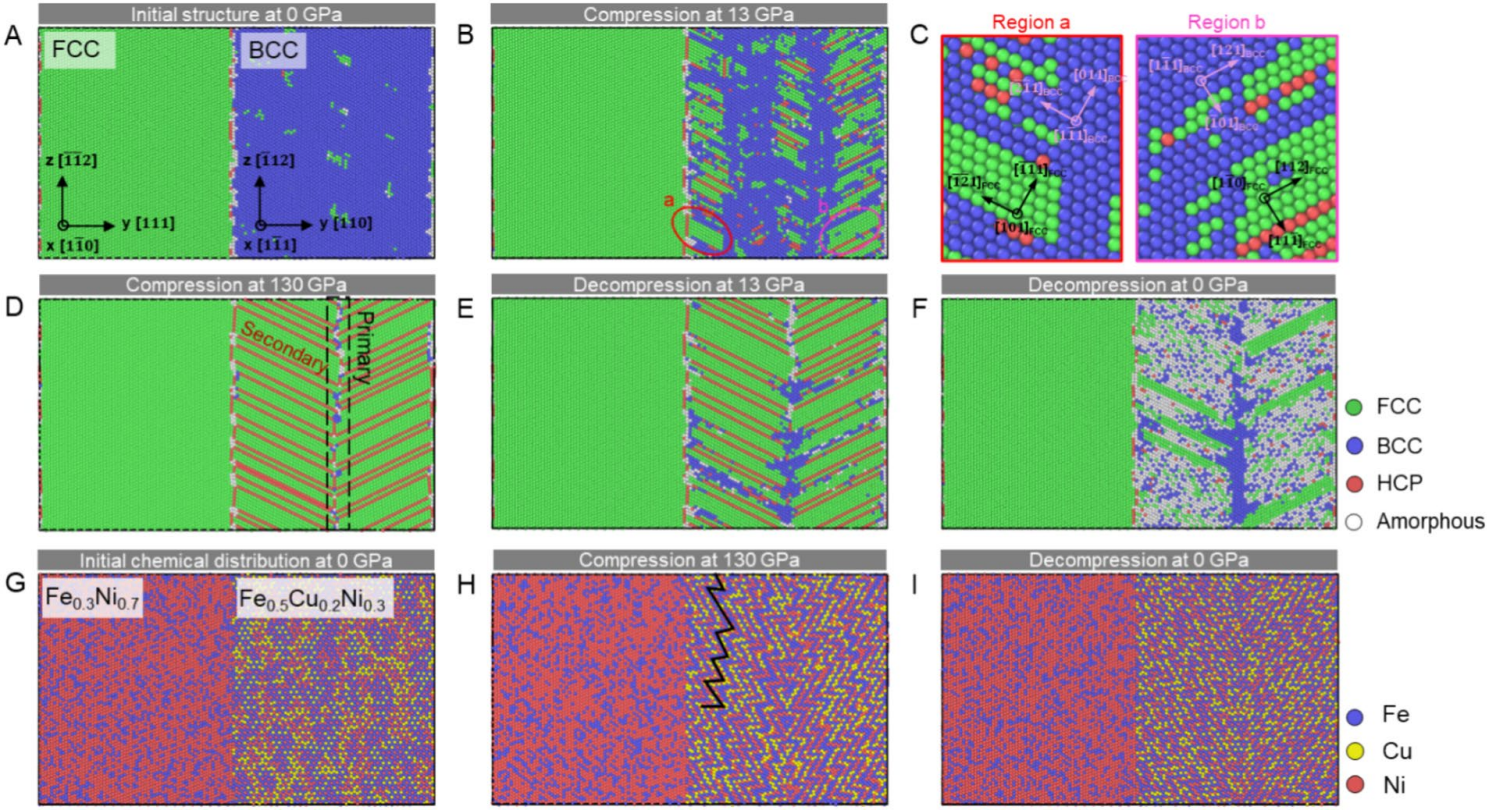
Atomistic simulation of phase transformation evolution in dual-phase model. The atomic structure at (**A**) initial state, (**B**) compressed at 13 GPa, (**D**) at 130 GPa, (**E**) decompressed to 13 GPa, and (**F**) to 0 GPa. (**C**) The relative orientation between the initial BCC phase and transformed FCC phase during BCC to FCC transformation. (**G**–**I**) Chemical mappings at (**G**) initial state, (**H**) 130 GPa, and (**I**) 0 GPa.

We perform MD simulations of dual-phase alloy structure to understand the process underlying BCC to FCC phase transformation. The initial structure is created to have the K–S orientation, as shown in Fig. 5. According to reference^18^, the ordered B2 structure can be formed in the as-printed sample. Considering that either Al or Ni typically favors the FCC phase, it is reasonable to hypothesize that the disruption of the B2 ordered structure during the BCC to FCC phase transition may stabilize the resulting FCC phase, leading to irreversibility. Furthermore, it is expected that the B2 ordered structure would be more susceptible to disruption with increased chemical complexity. To test this hypothesis, the following criteria for the potentials were established: (1) They must involve at least three elements; (2) The B2 structure should be representable; (3) Phase transformation should be achievable under high-pressure conditions. Through meticulous screening, a many-body embedded atom method (EAM) potential for the FeCuNi alloy was selected for employment in the molecular dynamics simulations.

The chemical composition (Fig. 5A) is different in two phases to reflect the difference of chemical composition as in experiment. In BCC phase, the B2 FeCu is created with 60% of Cu being replaced by Ni to capture the chemical complexity. With increasing the pressure to 13 GPa, the BCC to FCC phase transition occurs in the interface as well as the BCC interior, suggesting a heterogeneous transformation feature (Fig. 5B). Examining the cryptographic orientation in FCC and BCC structure (Fig. 5C), we observe two types of orientation pair, i.e., {111}FCC || {110}BCC and < 110 > FCC ||< 111 > BCC. At 130 GPa (Fig. 5D), the transformation completes and elucidates an FCC structure consisting of twin boundaries. Notably, the initial interphase boundary between BCC and FCC does not move during the transformation, which is in agreement with our high-pressure experiments. With decreasing the pressure (Fig. 5E, F), overall transformed FCC structure retains, even if some BCC phase emerges. The chemical distribution for initial state, highest pressure transformed state, and pressure-recovered states are shown in Fig. 5G–I, respectively. The solid line Fig. 5H highlighting the transformed FCC structure follows the K–S transition path^31^. Figure S4 shows the detailed effect of B2 order on the transformation irreversibility. With decreasing the degree of B2 order and reducing the stability of BCC phase, the reverse transform from FCC back to BCC becomes less likely to occur.

**Figure 6:**
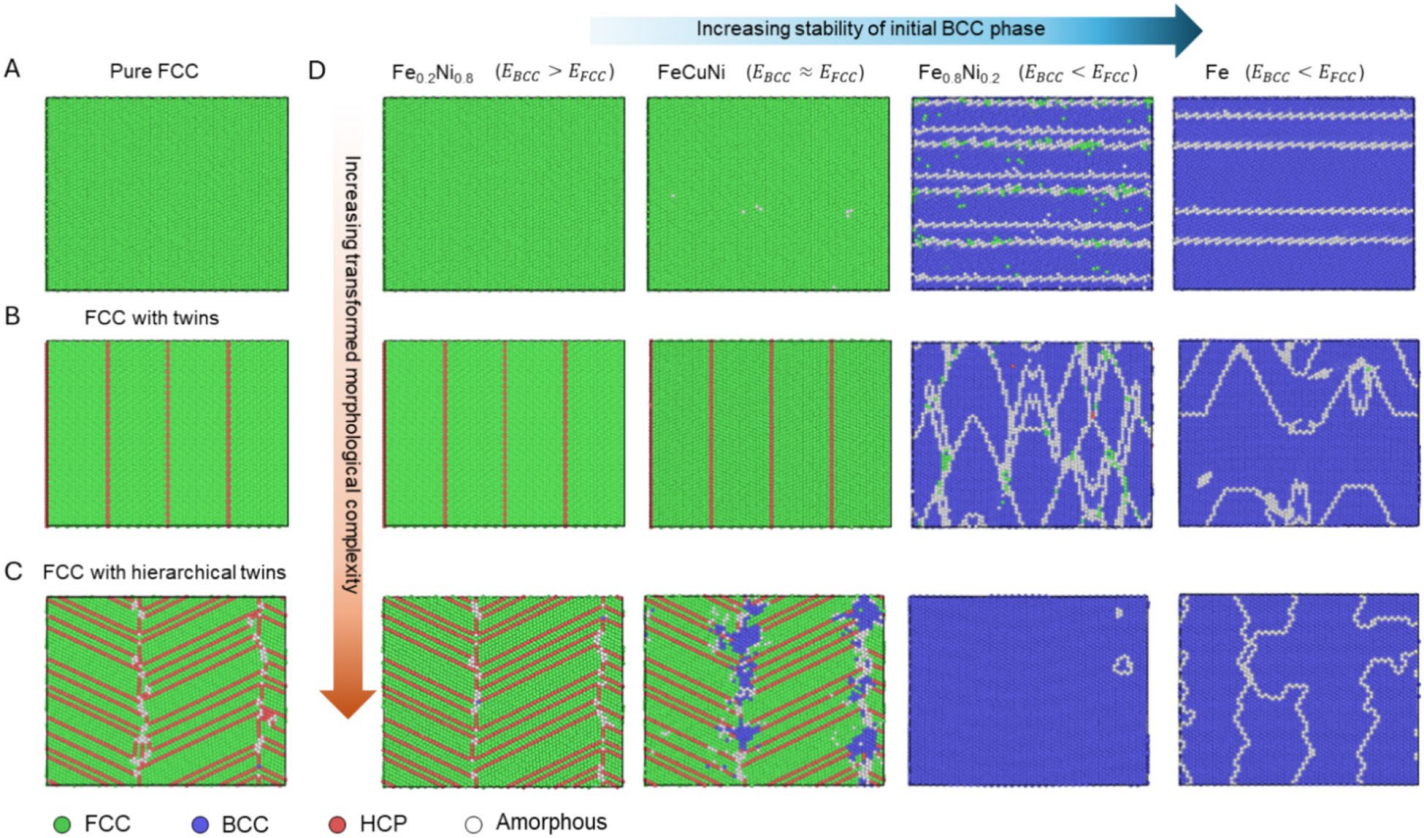
The influence of BCC phase stability and transformed morphological complexity on BCC to FCC recovery as decreasing pressure to 0 GPa. (**A**–**C**) shows the FCC morphology with perfect FCC structure, (**B**) with twins, and (**C**) with hierarchical twins, at 130 GPa. (**D**) The shows the structures after the pressure is decreased to 0 GPa. Four systems, including Fe_0.2_Ni_0.8_, FeCuNi, Fe_0.8_Ni_0.2_, and Fe, have been studied and shown.

Figure 6 provides a summary of the influence of BCC phase stability and transformed FCC structural heterogeneity on the irreversibility. Figure 6A–C shows FCC structures at 130 GPa with complexity, i.e., pure FCC phase, FCC phase with twins, and FCC phase with hierarchical twins, respectively. Figure 6D shows the structure after reducing the pressure to 0 GPa. It is noted that the horizonal direction indicates four systems studied, including Fe_0.2_Ni_0.8_, FeCuNi, Fe_0.8_Ni_0.2_, and pure Fe, which shows the increase in BCC stability. At the recovered pressure of 0 GPa, both Fe_0.8_Ni_0.2_ and Fe reveal reversible transformation from FCC to BCC. However, the Fe_0.2_Ni_0.8_ and FeCuNi maintain the FCC structure, indicating the relative phase stability between FCC and BCC dictates the reversibility. For FeCuNi with complex structure, the zero-pressure condition reveals partial nucleation of BCC clusters, implying that morphological complexity and defect sites promote BCC formation.

## Discussion

Table S2 shows the elemental analysis by Energy Dispersive Spectroscopy (EDS) of the as-printed EHEA Ni_40_Co_20_Fe_10_Cr_10_Al_18_W_2_ for both BCC and FCC phases. The comparison shows that in the as-printed sample of this EHEA, BCC-phase is rich in elements Ni and Al, while the FCC phase is rich in Co, Cr, Fe, and W. Table S3 shows the elemental analysis by EDS of the pressure-recovered sample where BCC nanolamellae has wholly transformed to FCC-phase and the composition is displayed for as-printed FCC phase and pressure-formed FCC phase. The comparison of elemental compositions in Table S2 and Table S3 clearly shows that within experimental errors the composition of the pressure-formed FCC phase has the same composition as the BCC phase in the as-printed sample. This clearly establishes that the BCC–FCC transformation is diffusionless.

In prior research, computational work has shown the BCC → FCC transition at high temperature^32,33^, uniaxial stretch^34,35^ or shock^36^. The irreversibility of BCC → FCC transition is attributed to the change of crystal symmetry during the phase transition^37^. Irreversibility is inevitable when the energy barrier to plastic deformation (e.g., twinning or slip) is no higher than the barrier to the phase transition itself. However, the BCC → FCC transition of HEA under hydrostatic pressure has rarely been discussed. To investigate the atomistic process of BCC → FCC phase transition, it would be best to adopt the potential for EHEA Ni_40_Co_20_Fe_10_Cr_10_Al_18_W_2_. However, due to limited choice of potentials, we could not find the right potential to simulate phase transformation under high pressure of this EHEA. An alternative system is required to simulate the BCC → FCC phase transition, meanwhile capturing the structural and chemical features of BCC phase in EHEA. In this study, we demonstrate the BCC → FCC transition in dual phase FeCuNi ternary alloy. Our results reveal that the irreversibility of BCC → FCC transition depends on the stability of initial BCC phase and transformed morphological complexity. We find that the decreased stability of initial BCC phase can enhance the irreversibility. In our FCC/BCC dual phase model, B2 structure with chemical complexity becomes less stable at ambient condition as small amount of FCC structure appears in the BCC phase (Fig. 6A). After BCC → FCC transition at 130 GPa, part of FCC phase retained after the pressure is removed, showing a certain degree of irreversibility. The decreased transformed morphological complexity can also enhance the irreversibility. Amorphous phases along the phase boundaries and primary twin boundaries facilitate the generation of BCC phases. In addition, edge dislocations on the boundaries can trigger phase transformation as well^38^. A defect-free transformed FCC phase makes it tough for the nucleation of BCC phase. From our simulation results, a possible BCC → FCC transition process in EHEA Ni_40_Co_20_Fe_10_Cr_10_Al_18_W_2_ is as follows: Under hydrostatic pressure, the dislocations inside the initial BCC/B2 phase triggers the BCC → FCC transition, creating a dislocation-less FCC phase^38^. Due to the breaking of B2 structure, the FCC phase becomes more energetically favorable than the BCC phase and, together with dislocation-less FCC phase, restricts the FCC → BCC transition during decompression.

## Conclusions

We have conducted a high-resolution imaging study on 3-D printed eutectic high-entropy alloy Ni_40_Co_20_Fe_10_Cr_10_Al_18_W_2_ showing nanolamellar BCC and FCC phases at ambient condition. Our x-ray diffraction studies in a diamond anvil cell reveal that the BCC–FCC phase transition completed at 9 GPa under high pressure is irreversible, and the sample can be retrieved after high pressures to perform high-resolution transmission electron microscopy. Nano diffraction studies conducted on nanolamellae conclusively prove that BCC layers completely transform to FCC under high pressures. The x-ray spectroscopy analysis for the nanolamellae indicates that the chemical compositions of nanolamellae are unaffected by the BCC–FCC phase transformation and the transformation proceeds by a diffusionless mechanism. The detailed lattice imaging on the pressure-formed FCC and the original FCC nanolamellae show that many variants of the FCC grains are formed from the BCC nanolamellae under high pressure. This results in a reduction of the grain size in pressure-formed FCC nanolamellae and precluding any specific crystallographic relations with the as-printed FCC nanolamellae. Molecular dynamics studies confirm the irreversibility of BCC–FCC transformations in this additively manufactured eutectic high-entropy alloy Ni_40_Co_20_Fe_10_Cr_10_Al_18_W_2_.

## Methods

### Sample fabrication

The EHEA samples used in this work were additively manufactured using an M290 (EOS) laser-powder bed fusion machine with an ytterbium-fiber laser (maximum power of 400 W and focal diameter of 100 μm). The starting powders were gas atomized Ni_40_Co_20_Fe_10_Cr_10_Al_18_W_2_ EHEA powders from Vilory Advanced Materials Technology. The particle size was varied from 15 to 53 µm with a mean size of 35 µm. The weight fractions of the FCC and BCC solid solution are 67% and 33%, respectively. See ref^18^ for more detail of the sample preparation and characterization. The manufactured EHEA samples were laser machined in to size suitable for static high-pressure studies in a diamond anvil cell.

### High-pressure studies

The EHEA Ni_40_Co_20_Fe_10_Cr_10_Al_18_W_2_ sample was studied in a diamond anvil cell under quasi-hydrostatic pressure utilizing a liquid pressure medium along with a ruby pressure calibrant. The sample was pressurized to 30 GPa and in-situ angle dispersive x-ray diffraction spectrum were recorded at beamline 16-ID-B, HPCAT, Argonne National Laboratory. The pressures were generated in a symmetric diamond anvil cell with a diamond culet size of 300 microns and a sample size of 50 microns in diameter and 30 microns in thickness. The experiment employed a liquid pressure medium for a quasi-hydrostatic environment consisting of 4:1 methanol:ethanol mixture and ruby fluorescence as a pressure marker. The synchrotron x-ray radiation with wavelength 0.4246 Å was used in the experiment at 16-ID-B, and the sample-to-detector distance of 313 mm was calibrated with a CeO_2_ sample.

### High-resolution imaging

Scanning electron microscopy (SEM) and transmission electron microscopy (TEM) analyses were conducted to investigate the composition, morphology, and atomic structure of printed and compressed EHEA samples. Specifically, a Helios G4 UX instrument (Thermo Fisher Scientific), equipped with a dual electron/ion beam system, was used to acquire secondary-electron and backscattering SEM images and to prepare ~ 50 nm thin slices for cross-sectional TEM measurements. Before microscopy measurements, all samples immersed in epoxy resin were polished carefully with Premium 1200 SiC abrasive paper (1200 grit), while an extensive amount of lubricant was used to minimize the polishing-induced damage. Then, alumina (particle sizes of 1 and 0.3 mm) and silica (particle size 0.05 mm) abrasive pasts were used to obtain mirror-finished surfaces for imaging. The samples were then coated with an iridium layer (2.0 nm thick) to create a conductive layer between the alloys’ surfaces and connect the samples with aluminum SEM stubs.

Thin TEM specimens were prepared for the TEM analysis from bulk samples according to the procedure reported earlier^39^. A 1 μm thick platinum layer was deposited onto a selected rectangular area of 10 μm × 0.5 μm. Approximately 5 μm deep trenches (with a 52◦ base angle) were milled by a gallium beam on both sides of the platinum layer (accelerating voltage: 5 keV, milling current: 27 nA). Then, the slice was lifted from the sample and polished with a gallium ion beam to about 50 nm thickness under an accelerating voltage of 5 keV and a milling current of 700 pA. In this way, a clean cross-section of the samples for TEM images was produced without milling artifacts.

Aberration-corrected scanning TEM (STEM) Spectra 30–300 (Thermo Fisher Scientific) operated at 300 keV (50 pm resolution in STEM mode) was used for the high-resolution imaging of the thin specimens lifted from the samples. Bright-field TEM and selected area electron diffraction (SAED) imaging were performed using a Ceta CMOS camera. Energy dispersive X-ray spectroscopy (EDS) elemental analysis/mapping was performed by utilizing Super-X EDS detection system using four silicone drift detectors. High-angle annular dark-field (HAADF) and bright-field (BF) STEM imaging were performed by a Panther STEM detection system. Nano diffraction was performed in STEM mode using a Ceta CMOS camera and TIA imaging software.

### Simulation model

To investigate the BCC → FCC phase transition, a nanolamellar FCC/BCC dual phase model of 600,000 atoms. In the FCC bulk, orientations [$1\overline{1 }0$], [$111$] and [$\overline{1 }\overline{1 }2$] were aligned along the x, y, and z directions, respectively. The build had orientations [$1\overline{1 }1$], [$1\overline{1 }0$] and [$\overline{11 }2$] aligned along the x, y, and z directions, respectively. The orientation relationship of FCC bulk and BCC bulk adhere to the K–S relationship^31^. The boundary of the FCC/BCC dual phase model lies in the x–z plane. The chemical composition, unit length and model box length of FCC and BCC bulk are listed in the following Table 1.

**Table 1:** Chemical composition, unit length and box length of dual phase models.

	Structure	Chemical composition	Unit length (Å)			Box length(Å)		
			x	y	z	x	y	z
Model 1	FCC	Fe_0.3_Ni_0.7_	2.482	6.079	4.299	156.36	133.75	159.10
	B2	Fe_0.5_Cu_0.2_Ni_0.3_	4.890	3.993	6.916	156.49	139.76	159.07
Model 2	FCC	Fe_0.3_Ni_0.7_	2.482	6.079	4.299	158.84	133.75	167.65
	B2	FeCu	4.967	4.039	6.996	158.29	141.36	167.89
Model 3	FCC	Fe_0.3_Ni_0.7_	2.482	6.079	4.299	158.84	133.75	167.65
	BCC	Fe_0.5_Cu_0.2_Ni_0.3_	4.930	4.025	6.972	157.75	140.88	167.32
Model 4	FCC	Fe_0.3_Ni_0.7_	2.482	6.079	4.299	158.84	133.75	167.65
	BCC	Fe_0.28_Cu_0.36_Ni_0.36_	4.931	4.026	6.973	157.78	140.90	167.34

To minimize residual stress arising from the lattice misalignment, the box length of FCC and BCC bulks along the x and z directions were closely matched.

A many body EAM potential was employed for a FeCuNi alloy^40^. Periodic boundary conditions were applied on three dimensions of model. We used the method of energy minimization by conjugating gradients on the EAM model to relax the residual stress in the interface. The model was thermally equilibrated at 300 K by an isotropic zero-pressure isobaric-isothermal NPT ensemble for 10 ps with a molecular dynamic time step of 0.001 ps. After relaxation, an isotropic pressure was applied to the model with a constant rate of 0.65 GPa/ps up to 130 GPa. And then zero pressure condition was achieved by decreasing the pressure at 0.65 GPa/ps down to atmospheric pressure. The Large-scale Atomic/Molecular Massively Parallel Simulator (LAMMPS) was utilized to carry out the simulation^41^, and atomic structures were visualized by the molecular visualization package OVITO^42^.

### Supplementary Information

Supplementary Information.

## Data Availability

Data is provided within the manuscript or supplementary information files.
