# Supplementary material for: High-pressure phase transition in 3-D printed nanolamellar high-entropy alloy by imaging and simulation insights
Source: Sci Rep. 2024 Jul 16;14:16472. doi: 10.1038/s41598-024-67422-x (PMC11252327; doi:10.1038/s41598-024-67422-x)
Supplement: Supplementary file 1 — Supplementary Information. [file 41598_2024_67422_MOESM1_ESM.docx]

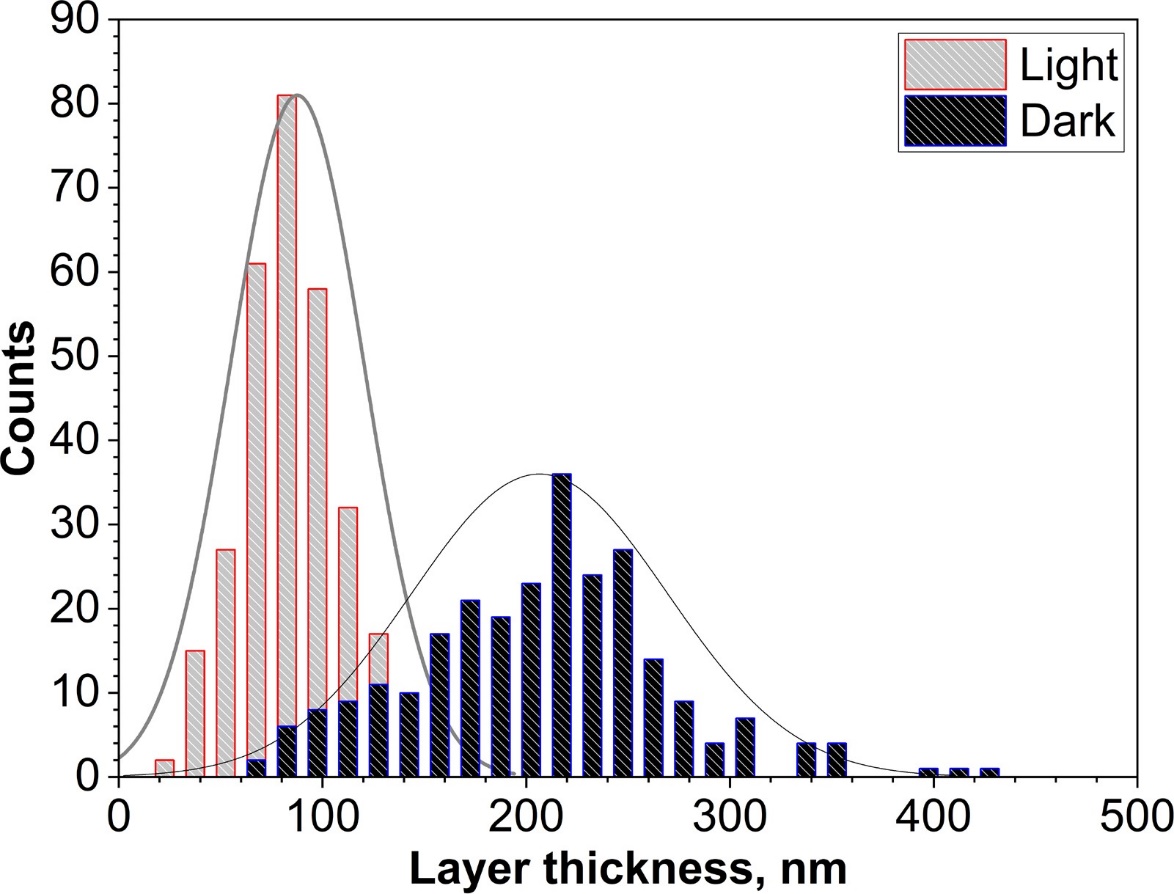


**Figure S1:** The measured nanolamellae thickness distribution for BCC (Light) and FCC (Dark) in the as-printed EHEA Ni_40_Co_20_Fe_10_Cr_10_Al_18_W_2_.


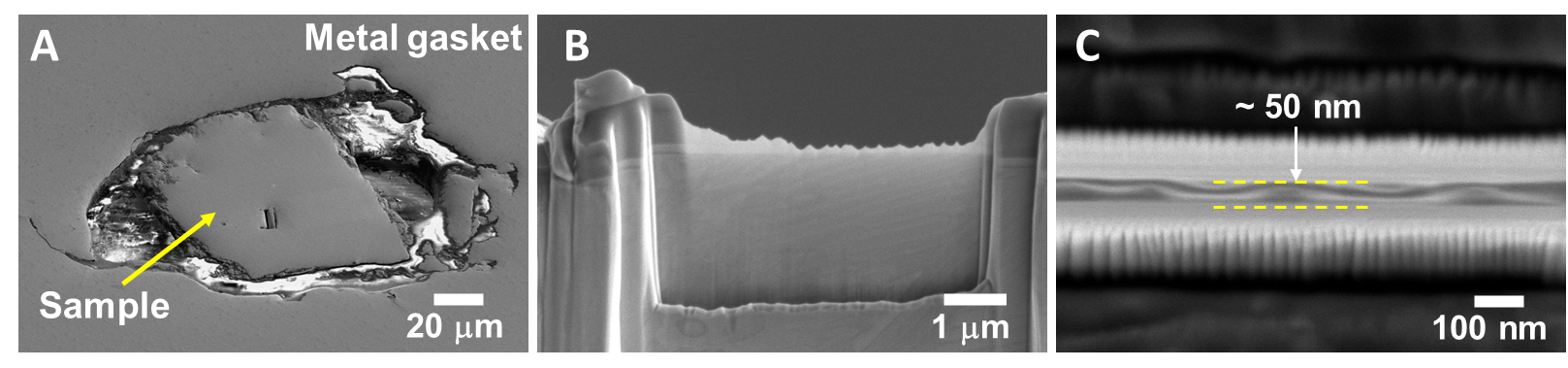


**Figure S2**: Ni_40_Co_20_Fe_10_Cr_10_Al_18_W_2_ EHEA sample at the center of the metal gasket after diamond anvil cell compression (A), side (B), and top (C) views of TEM specimen lifted out from the sample using gallium-ion milling method.

**Table S1:** Measured lattice spacings from SADP in Figure 3B and simulated spacings for FCC and BCC phases.

| **Measured lattice spacings, nm** | **Simulated lattice spacings, nm** | |
| --- | --- | --- |
|  | **FCC (a=0.3591 nm)** | **BCC (a=0.2872 nm)** |
| 0.208 | 0.206 (111) | 0.203 (110) |
| 0.127 | 0.126 (220) | 0.128 (210) |
| 0.114 | 0.113 (310) | 0.117 (211) |
| 0.108 | 0.108 (311) |  |
| 0.104 | 0.104 (222) | 0.102 (220) |
| 0.086 | 0.086 (322) | 0.087 (311) |
| 0.080 | 0.080 (420) | 0.080 (320) |
| 0.073 | 0.073 (422) | 0.072 (400) |


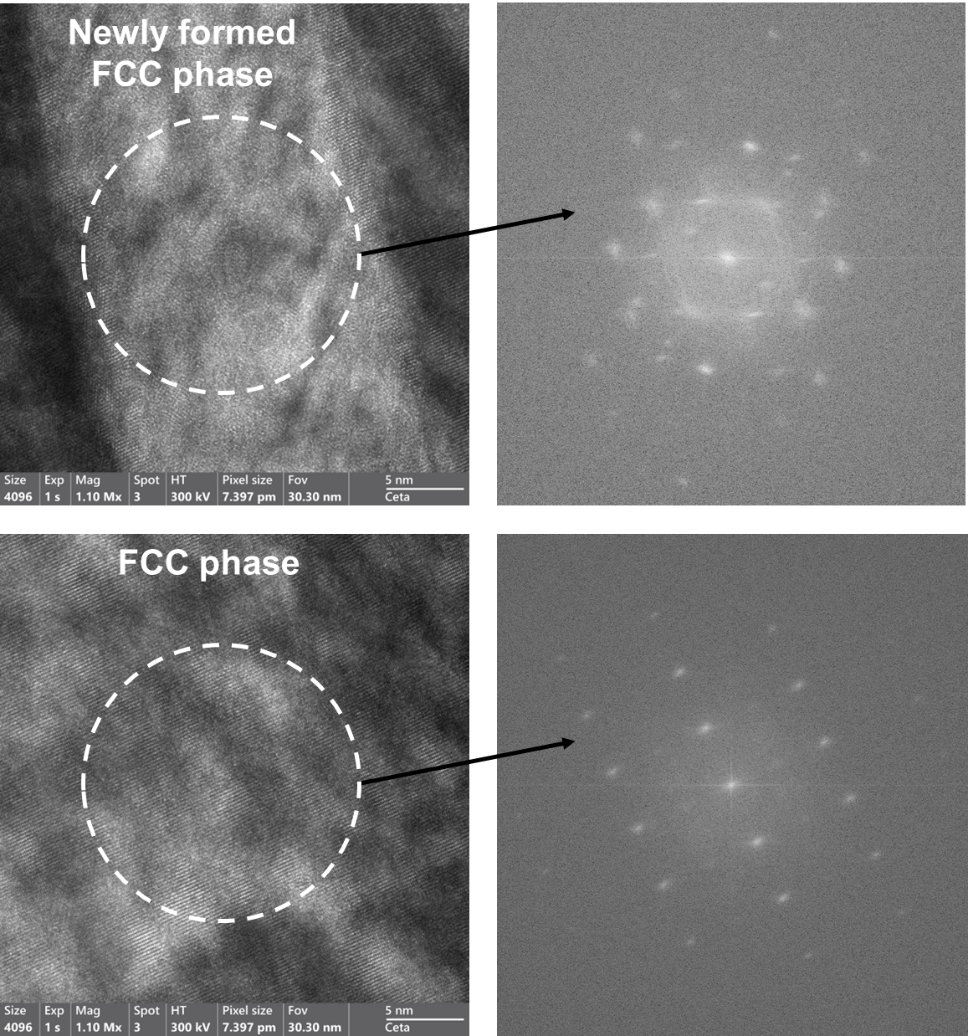


**Figure S3**: High-resolution TEM and Fast Fourier Transform (FFT) images of two FCC phases in the recovered sample.

**Table S2:** Elemental analysis of as-printed EHEA Ni_40_Co_20_Fe_10_Cr_10_Al_18_W_2_ for both BCC and FCC phases

| **Element** | **BCC-Phase (atomic %)** | **FCC-Phase (atomic %)** |
| --- | --- | --- |
| Al | 27.41 ± 1.93 | 10.83 ± 0.76 |
| Cr | 4.16 ± 0.61 | 12.39 ± 1.67 |
| Fe | 6.94 ± 0.99 | 11.50 ± 1.56 |
| Co | 15.44 ± 2.02 | 22.96 ± 2.76 |
| Ni | 45.20 ± 3.79 | 39.75 ± 3.83 |
| W | 0.85 ± 0.10 | 2.57 ± 0.31 |

**Table S3:** Elemental analysis of pressure recovered EHEA Ni_40_Co_20_Fe_10_Cr_10_Al_18_W_2_ sample for the two FCC phases.

| **Element** | **FCC-Phase (atomic %)**  **(pressure formed)** | **FCC-Phase (atomic %)**  **(as printed)** |
| --- | --- | --- |
| Al | 23.07 ± 1.74 | 9.58 ± 0.71 |
| Cr | 3.89 ± 0.58 | 10.84 ± 1.51 |
| Fe | 7.22 ± 1.04 | 11.42 ± 1.58 |
| Co | 16.55 ± 2.19 | 22.32 ± 2.75 |
| Ni | 48.40 ± 3.86 | 43.69 ± 3.95 |
| W | 0.88 ± 0.11 | 2.15 ± 0.27 |


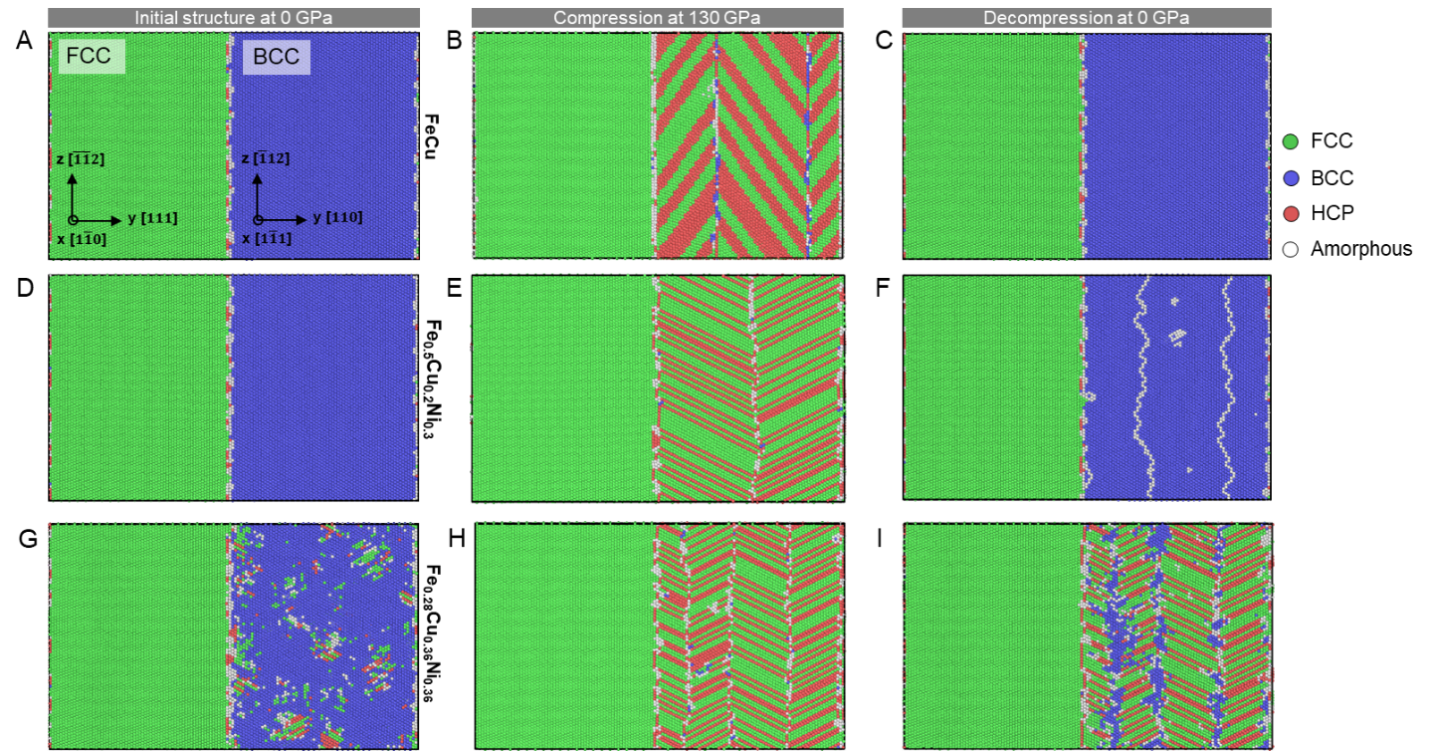


**Figure S4:** Atomistic simulation of BCC → FCC transition in different dual phase models. (A-C) BCC phase is FeCu B2 structure. Atomic slides show structural distribution at (A) initial state, (B) compressed at 130 GPa, (C) decompressed at 0 GPa. (D-F) BCC phase is RSS Fe_0.5_Cu_0.2_Ni_0.3_. (G-I) BCC phase is RSS Fe_0.5_Cu_0.2_Ni_0.3_.
